# Ultrasonographic assessment of entheseal sites of upper and lower extremities in hemodialysis patients using Madrid Sonography Enthesitis Index

**DOI:** 10.1186/s12891-022-05512-5

**Published:** 2022-06-23

**Authors:** Reham Sabry, Samar Tharwat, Mohammed Kamal Nassar, Ehab E. Eltoraby

**Affiliations:** 1 Nephrology unit, Internal Medicine Department, Mansoura New General Hospital, Mansoura University, Mansoura, Egypt; 2 Rheumatology & Immunology Unit, Internal Medicine Department, Faculty of Medicine, Mansoura University, El Gomhouria St, Mansoura, Dakahlia Governorate 35511 Egypt; 3 Mansoura Nephrology & Dialysis Unit (MNDU), Department of Internal Medicine, Faculty of Medicine, Mansoura University, Mansoura, Egypt

**Keywords:** Ultrasonography, Enthesitis, Enthesopathy, Hemodialysis, MASEI score

## Abstract

**Background:**

There is no much information about the entheseal involvement among hemodialysis (HD) patients. The aim of this study was to assess the frequency and distribution of ultrasonographic (US) entheseal alterations in HD patients and to evaluate the association between US abnormalities and both clinical and laboratory data.

**Methods:**

This study was conducted on 41 HD patients and 23 sex- and age- matched controls. All participants were evaluated clinically for any signs of enthesopathy. Six entheses sites were scanned bilaterally using grey scale (GS) and power Doppler (PD) US and were scored using Madrid Sonography Enthesitis Index (MASEI) scoring system.

**Results:**

In HD patients, at least one clinical sign suggestive of enthesopathy was found in 69 (14%) of 492 entheses. HD patients had statistically significant higher scores of structural tendon abnormalities (*p* < 0.001), enthesis thickening (*p* < 0.001), bone erosions (*p* < 0.001) and calcification (*p* = 0.037) than the healthy controls. Total MASEI score was higher in HD patients than healthy controls (median;18 vs 8, *p* < 0.001), also, MASEI-inflammatory (median;11 vs 3, *p* < 0.001) and damage scores (median;6 vs 0, *p* < 0.001). There was a statistically significant positive association between total MASEI score and both age (*p* = 0.032) and duration of HD (*p* = 0.037). Duration of HD was predictive for both MASEI-damage component (*p* = 0.004) and total MASEI score (*p* = 0.014).

**Conclusion:**

There is a high prevalence of subclinical enthesopathy in HD patients. The entheseal US alterations is much higher in HD patients than in healthy subjects. The duration of HD is the significant predictor of enthesopathy in HD patients.

## Introduction

Chronic kidney disease (CKD) affects about 10–15% of adults worldwide [1]. And therefore, end stage kidney disease (ESKD) represents a global-health and health-care burden that is rapidly increasing [2]. The high prevalence of diabetes mellitus, hypertension, obesity, and ageing are all contributing to the global rise in this condition [3]. Dialysis is a form of renal replacement therapy that ensures maintenance of homeostasis in patients with ESRD [4]. Currently, dialysis is life-saving to nearly 3.4 million people with ESRD around the world [5]. Hemodialysis (HD), peritoneal dialysis, and hemofiltration are the three main forms of dialysis [6].

Although HD can reduce morbidity and mortality in renal patients, it can also cause a number of complications during dialysis sessions and/or as a result of long-term HD use [7]. Amyloidosis, bone disease, endocrine abnormalities, infection and cardiovascular complications are all risks associated with long-term HD [7]. Musculoskeletal (MSK) complications are one of the most common health concerns that impact individuals on maintenance HD [8]. Bone cysts, destructive arthropathy, amyloidosis, carpal tunnel syndrome, and spontaneous tendons rupture are some of these MSK complications [9, 10].

In HD patients, spontaneous ruptures of the Achilles and/or quadriceps tendon have been documented on a sporadic basis and are usually viewed as isolated events [11]. The reasons that predispose to such ruptures are still unknown. Some researchers believe that the tendon is still intact and that the rupture is caused by osteolytic bone resorption at the enthesis [12]. Enthesis is the area where ligaments, tendons, or joint capsules attach to bone [13].

Ultrasonography (US) is a widely available, noninvasive, reproducible, low-cost imaging modality that does not expose patients to ionizing radiation and can be used to diagnose, monitor, and stratify enthesopathy. When it comes to diagnosing enthesopathy, US is more sensitive than clinical examination, demonstrating a high frequency of abnormal findings even in asymptomatic patients [14]. However, little information is known about the entheseal involvement in HD patients; only few studies have been carried out to assess if HD patients have a ‘subclinical’ enthesopathy [15, 16]. In some clinical contexts, such as in cases of “difficult to treat” pain syndromes, an early diagnosis of entheseal involvement may be crucial. Therefore, the aim of this study was to evaluate the frequency and distribution of entheseal US alterations in HD patients and to evaluate the association between US findings and both clinical characteristics and laboratory data.

## Materials and methods

### Study population

This cross-sectional observational study was carried out from October 2017 to March 2020 on 41 patients with ESRD and receiving HD at Mansoura Nephrology and Dialysis Unit (MNDU), Mansoura University Hospital, Egypt. The sample size was selected as a convenience sample. All patients who met the inclusion criteria were offered to participate in the study unless they qualify for any of the exclusion criteria or refuse to participate. Inclusion criteria included ESRD patients aged more than 18 years and undergoing HD for more than 6 months, three times per week. Patients who had a history of rheumatic or autoimmune diseases, diabetes mellitus, peripheral neuropathy, chronic use of steroids and/ or fluoroquinolone therapy during the previous 6 months, sever trauma or joint surgery were excluded from the study. Twenty-three age- and sex matched healthy controls were also included. The study was performed in accordance with the Declaration of Helsinki. It was approved by the Institutional Research Board of the Faculty of Medicine, Mansoura University (Approval number: MS/17.05.120). The study was explained to all participants, and informed written consent was obtained from all of them before starting the study.

### Data collection

Demographic data including age, sex, occupation, and marital status were recruited. Body weight was measured. Other clinical parameters (e.g., vascular access side and HD duration) were obtained from each HD patient through an interview.

### Clinical evaluation of different entheseal sites

Clinical evaluation was conducted to all HD patients and healthy controls by an expert rheumatologist to detect any clinical signs indicative of enthesopathy. These signs included swelling or tenderness elicited by mobilization, pressure,and contraction against resistance of the corresponding entheses.

### Madrid sonographic Enthesis index (MASEI) scoring system

All US examinations were carried out by a rheumatologist with at least 7 years of experience in the field of musculoskeletal ultrasound (MSUS). At the time of US evaluation, the rheumatologist was blind to the clinical assessment of the entheseal sites. The EDAN U2 ultrasound equipment (Shenzhen, China) with a linear array transducer was employed in the study (8 to 13.4 MHz). The frequency was set to 13 MHz, and the sonographic settings were adjusted to provide the best images of the scanned enthesis. With a constant room temperature of 21 C and a lower wall filter, power Doppler (PD) settings were standardized with a pulse repetition frequency (PRF) of 0.75–1.20 kHz and a PD gain of 50–55 dB.

Six entheses were scanned bilaterally in axial and longitudinal planes as specified by de Miguel [17]. The scanned entheseal sites included the following: proximal plantar aponeurosis, distal Achilles’ tendon, distal and proximal patellar ligament insertion, distal quadriceps tendon and distal triceps tendon. Entheses were scored using the Madrid Sonography Enthesitis Index (MASEI) scoring system [17]. The elementary lesions included thickness, structural tendon abnormalities, enthesis thickening, bone erosions, calcification, PD and bursitis. Tendon structure was defined as abnormal if hypoechoic aspect, loss of fibrillar pattern or fusiform thickening of the enthesis occurred. Thicknesses of ligaments, tendons, and fascia were determined on the axial scan as the maximum anteroposterior diameter in millimeters, excluding the paratenon at the point of maximum thickness proximal to the bony insertion.

Additionally, analysis 2 subscores of MASEI were included; the MASEI-inflammatory (structural tendon abnormalities, enthesis thickening, PD and bursitis) and MASEI-damage (calcifications, enthesophytes, and erosions) [18].

### Blood sampling and laboratory tests

Just before starting the first HD session of the week, blood samples were taken from the vascular access of HD patients. An automated analyzer was used to perform laboratory tests on the days of blood sampling. Laboratory tests included complete blood count (CBC), total cholesterol, triglycerides, serum calcium, phosphorus, parathyroid hormone level (PTH) and uric acid.

### Statistical analysis

Data were collected, and analyzed using IBM SPSS Statistics version 24.0 for Windows (IBM Corp., Armonk, NY, USA) on a personal computer. For all quantitative values, medians (interquartile range [IQR] or 25th percentile – 75th percentile) or means ± standard deviation were used, while numbers of cases and percentages (%) were utilized to illustrate qualitative variables. The Shapiro-Wilk test was used to check for normality in the distribution of continuous variables and the significance of differences between the two groups was assessed by the independent samples t test for normally distributed variables or by the Mann-Whitney test for non-parametric variables. For comparisons between qualitative variables, Chi-square or Fisher exact tests were utilized, as appropriate. An analysis of covariance (ANCOVA) test was used to determine the effect of HD on MASEI score assuming age as a covariate. Correlation analysis using the Spearman’s test were used to define relevant factors that affected MASEI scores. Subsequently, univariate linear regression analysis was done to assess the predictors of MASEI scores. *P* value less than 0.05 was considered significant.

## Results

### Study population

The study included a total of 41 HD patients (mean age 50.7 ± 14.5 years, 25 males and 16 females) and 23 healthy sex- and age-matched controls (mean age 45.3 ± 8.9 years, 14 males and 9 females). We scanned 492 entheses sites in HD patients and 276 in healthy controls. Table 1 shows the clinical characteristics and laboratory data of HD patients and healthy controls. For the HD group, the median duration since onset of HD was 4 years. Majority of patients (80.5%) had vascular access at the right side.

**Table 1:** Clinical characteristics and laboratory data of HD patients (*n* = 41) and healthy subjects (*n* = 23)

Characteristic	HD patients (*n* = 41)	Healthy subjects (*n* = 23)	P
Age (years)	50.7 ± 14.5	45.3 ± 8.9	0.068
Sex			
Males	25 (61%)	14 (60.9%)	0.993
Females	16 (39%)	9 (39.1%)	
Body weight (Kg)	73 (63–78.8)	77 (70–80)	0.266
Occupation			
Not working	22 (53.7)	18 (78.3)	0.051
Working	19 (46.3)	5 (21.7)	
Marital status			
Single	9 (22)	10 (43.5)	0.053
Married	32 (78)	13 (56.5)	
Duration of HD (years)	4 (2–6)	–	–
Vascular access			
Right side	8 (19.5)	–	–
Left side	33 (80.5)	–	
Hemoglobin (g/dL)	9.9 (9.2–10.9)	N/A	–
Cholesterol (mg/dL)	110 (93–130.5)	N/A	–
Triglycerides (mg/dL)	73 (63–10.4.5)	N/A	–
Calcium (mg/dL)	8.5 (7–9.5)	N/A	–
Phosphorus (mg/dL)	6 (3.6–8.5)	N/A	–
PTH (pg/mL)	518 (207–1090.5)	N/A	–
Uric acid (mg/dl)	6.4 (5.4–7)	N/A	–

Quantitative variables are expressed as mean ± standard deviation for normally distributed data and median (25th percentile – 75th percentile) for non-normally distributed data. Qualitative variables are expressed as number (%)

*PTH* Parathyroid hormone

### Clinical evaluation of different entheseal sites

**Fig. 1:**
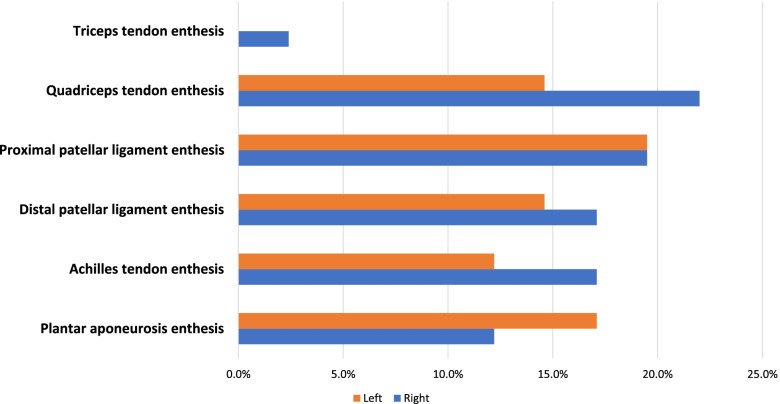
Clinical evidence of enthesopathy at different entheseal sites in HD patients (*n* = 41)

Clinical examination revealed at least one sign suggestive of enthesopathy in 69 (14.02%) of 492 entheses in 41 HD patients. Of which, the most frequently involved entheseal site was proximal patellar ligament (19.5%) followed by quadriceps tendon (18.3%) while the least involved was triceps tendon enthesis (2.4%). No clinical evidence of enthesopathy was detected in healthy controls. Figure 1 shows the distribution of entheseal sites that clinically involved in HD patients.

### Madrid sonographic Enthesis index (MASEI) scoring system

Table 2 illustrates the morphostructural changes of each enthesis according to MASEI scoring system. HD patients had significantly higher thickness and structural tendon abnormalities at almost all scanned entheseal sites when compared to healthy individuals.

**Table 2:** The morphostructural changes of each entheses according to Madrid Sonographic Enthesis Index (MASEI) Scoring System in HD patients (*n* = 41) and healthy subjects (*n* = 23)

Parameter	HD patients (*n* = 41) Entheses (82)	Healthy controls (*n* = 23) Entheses =46	P
Inferior pole of the calcaneus: plantar aponeurosis enthesis			
Thickness,mm, median (IQR)	4.3 (1.35)	3.15 (1.6)	0.000*
Stuctural tendon abnormalities,n (%)	46 (56.1)	14 (30.4)	0.005*
Enthesis thickening,n (%)	36 (43.9)	7 (15.2)	0.001*
Bone erosions,n (%)	5 (6.1)	1 (2.2)	0.418
Calcification,n (%)			
Mild	2 (2.4)	0	0.104
Moderate	14 (17.1)	3 (6.5)	
Severe	3 (3.7)	5 (10.9)	
Power Doppler signal,n (%)	0	0	–
Superior pole of the calcaneus: Achilles tendon enthesis			
Thickness,mm, median (IQR)	5.2 (1.53)	4.1 (1.3)	0.000*
Stuctural tendon abnormalities,n (%)	16 (19.5)	4 (8.7)	0.132
Enthesis thickening,n (%)	40 (48.8)	6 (13)	0.000
Retrocalcaneal bursitis	10 (12.2)	3 (6.5)	0.375
Bone erosions,n (%)	10 (12.2)	0	0..014*
Calcification,n (%)			
Mild	6 (7.3)	1 (2.2)	0.208
Moderate	8 (9.8)	2 (4.3)	
Severe	0	1 (2.2)	
Power Doppler signal,n (%)	0	0	–
Tibial tuberosity: distal patellar ligament enthesis			
Patellar ligament structure,n (%)	55 (67.1)	13 (28.3)	0.000*
Thickness,mm, median (IQR)	4.45 (1.6)	3.4 (0.85)	0.000*
Stuctural tendon abnormalities,n (%)	27 (32.9)	2 (4.3)	0.000*
Enthesis thickening,n (%)	50 (61)	10 (21.7)	0.000*
Infrapatellar bursitis,n (%)	4 (4.9)	2 (4.3)	1
Bone erosions,n (%)	7 (8.5)	1 (2.2)	0.257
Calcification,n (%)			
Mild	4 (4.9)	0	0.401
Moderate	2 (2.4)	1 (2.2)	
Severe	1 (1.2)	0	
Power Doppler signal,n (%)	0	0	–
Inferior pole of the patella: proximal patellar ligament enthesis			
Thickness,mm, median (IQR)	4.55 (1.23)	3.5 (1.33)	0.000*
Stuctural tendon abnormalities,n (%)	18 (22)	4 (8.7)	0.086
Enthesis thickening,n (%)	55 (67.1)	13 (28.3)	0.000*
Bone erosions,n (%)	4 (4.9)	1 (2.2)	0.654
Calcification,n (%)			
Mild	1 (1.2)	1 (2.2)	0.693
Moderate	1 (1.2)	0	
Severe	0	0	
Power Doppler signal,n (%)	0	0	–
Superior pole of the patella: quadriceps tendon enthesis			
Thickness,mm, median (IQR)	6.4 (1.7)	6 (1.53)	0.002*
Stuctural tendon abnormalities,n (%)	36 (43.9)	9 (19.6)	0.006*
Enthesis thickening,n (%)	56 (86.3)	19 (41.3)	0.003*
Bone erosions,n (%)	28 (34.1)	4 (8.7)	0.001*
Calcification,n (%)			
Mild	7 (8.5)	2 (4.3)	0.218
Moderate	11 (13.4)	2 (4.3)	
Severe	1 (1.2)	0	
Power Doppler signal,n (%)	3 (3.7)	0	0.552
Olecranon tuberosity: triceps tendon enthesis			
Thickness,mm, median (IQR)	4.05 (2.43)	2.7 (1.03)	0.000*
Stuctural tendon abnormalities,n (%)	15 (18.3)	1 (2.2)	0.010*
Enthesis thickening,n (%)	35 (42.7)	0	0.000*
Bone erosions,n (%)	12 (14.6)	1 (2.2)	0.031*
Calcification,n (%)			
Mild	6 (7.3)	0	0.091
Moderate	2 (2.4)	0	
Power Doppler signal,n (%)	1 (1.2)	0	1

*p<0.05

Table 3 shows the total MASEI scores of each component and each enthesis in HD patients and healthy subjects. There were statistically significant higher scores of structural tendon abnormalities (*p* < 0.001), enthesis thickening (*p* < 0.001), bone erosions (*p* < 0.001) and calcification (*p* = 0.037) in HD patients when compared with healthy subjects. Also, total MASEI score of each enthesis was significantly higher in HD patients in comparison to healthy subjects. HD patients had significantly higher total MASEI score than healthy controls (median;18 vs 8, *p* < 0.001), also higher MASEI-inflammatory (median;11 vs 3, *p* < 0.001) and damage scores (median;6 vs 0, *p* < 0.001). There was a significant effect of HD on the total and inflammatory MASEI sore (*p* = 0.012,0.011, respectively) after controlling for the effect of age.

**Table 3:** The total MASEI scores of each component and each enthesis in HD patients (*n* = 41) and healthy subjects (*n* = 23)

Enthesis	HD patients (*n* = 41) Entheses (82) median (IQR)	Healthy controls (*n* = 23) Entheses =46 median (IQR)	P
MASEI components score (maximum score)			
Stuctural tendon abnormalities (12)	4 (3)	1 (3)	0.000*
Enthesis thickening (12)	7 (3)	1 (3)	0.000*
Bursitis (4)	0 (0)	0 (0)	0.632
Bone erosions (36)	3 (6)	0 (0)	0.000*
Calcification (36)	2 (5)	0 (2)	0.037*
Power Doppler signal (36)	0 (0)	0 (0)	0.187
Total MASEI score at:			
Plantar aponeurosis enthesis	3 (4)	0 (4)	0.015*
Achilles tendon enthesis	2 (5)	0 (1)	0.003*
Distal patellar ligament enthesis	2 (3)	1 (1)	0.000*
Proximal patellar ligament enthesis	2 (2)	1 (1)	0.001*
Quadriceps tendon enthesis	4 (7)	1 (3)	0.000*
Triceps tendon enthesis	1 (3)	0	0.000*
Total MASEI score	18 (11.5)	4 (8)	0.000*
MASEI-inflammatory	11 (7)	3 (5)	0.000*
MASEI-damage	6 (9)	0 (2)	0.000*

Of a total 492 scanned entheseal sites in 41 HD patients, the highest number of elemental lesions seen was enthesis thickening (272/492,55.3%), followed by structural tendon abnormalities (158/492,32.1%), calcification (69/492,14%), bone erosions (66/492,13.4%), bursitis (14/492, 2.8%) and power Doppler signal (40/492,8%) as shown in Table 4.

**Table 4:** Frequency of involvement of MASEI score components in HD patients (*n* = 41) and healthy subjects (*n* = 23)

MASEI score components	HD patients (*n* = 41) Entheses =492 n (%)	Healthy controls (*n* = 23) Entheses =276 n (%)	P
Stuctural tendon abnormalities	158 (32.1)	34 (12.3)	0. 000*
Enthesis thickening	272 (55.3)	55 (19.9)	0. 000*
Bursitis	14 (2.8)	5 (1.8)	0.364
Bone erosions	66 (13.4)	8 (2.9)	0.000*
Calcification	69 (14)	18 (6.5)	.002*
Power Doppler signal	4 (0.8)	0	0.303
Presence of one sign of enthesopathy	339 (68.9)	85 (30.8)	0.000*

**Fig. 2:**
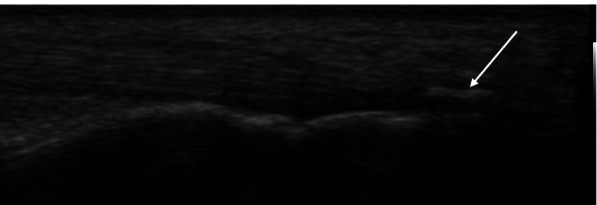
A 60-year-old female patient with chronic renal failure on chronic hemodialysis for 5 years with calcification at the posterior pole of calcaneus enthesis

**Fig. 3:**
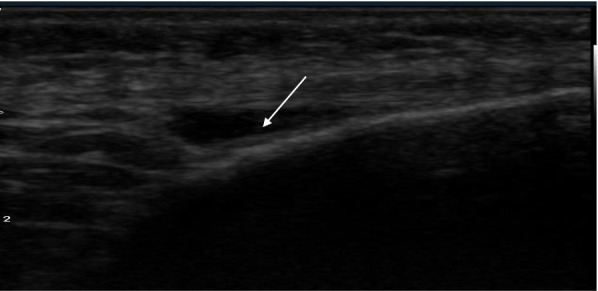
A 42-year-old male patient with chronic renal failure on chronic hemodialysis for 6 years with deep infrapatellar bursitis

Figure 2 illustrates calcification at the posterior pole of calcaneus enthesis detected in a HD patient and Fig. 3 shows deep infrapatellar bursitis in another HD patient.

### Correlation between MASEI scores and clinico-laboratory data

As shown in Table 5, there was a statistically significant positive association between total MASEI score and both age (*p* = 0.032) and duration of HD (*p* = 0.037). However, no statistically significant correlation was found between total MASEI, MASEI-inflammatory, and MASEI-damage sores and the following variables: body weight, total cholesterol, serum calcium, phosphorous and parathyroid hormone levels.

**Table 5:** Correlation between MASEI scores and clinico-laboratory data among HD patients (*N* = 41)

Variable	MASEI-inflammatory		MASEI-damage		Total MASEI	
	r_**s**_	P	r_**s**_	P	r_**s**_	P
Age (years)	0.297	0.059	0.261	0.099	0.336	0.032*
Sex	0.216	0.175	−0.064	0.693	0.055	0.733
Body weight (Kg)	0.128	0.427	−0.057	0.725	0.029	0.855
Duration of HD (years)	0.171	0.284	0.341	0.029	0.327	0.037*
Associated comorbidities	−0.052	0.745	0.099	0.539	0.029	0.856
Hemoglobin (g/dL)	−0.205	0.199	−0.240	0.130	−0.216	0.176
Cholesterol (mg/dL)	−0.275	0.105	0.128	0.424	−0.036	0.822
Triglycerides (mg/dL)	−0.106	0.510	−0.112	0.485	−0.134	0.403
Calcium (mg/dL)	−0.214	0.180	0.066	0.682	−0.082	0.610
Phosphorus (mg/dL)	0.047	0.770	0.284	0.072	0.139	0.385
PTH (pg/mL)	0.139	0.386	0.076	0.639	0.131	0.414
Uric acid (mg/dl)	0.106	0.511	0.147	0.358	0.138	0.388

*P < 0.05,PTH: Parathyroid hormone

### Predicting MASEI scores in HD patients

Univariate regression analyses revealed the following data: age was predictive for MASEI-inflammatory component (*p* = 0.042) while duration of HD was predictive for both MASEI-damage component (*p* = 0.004) and total MASEI score (*p* = 0.014) as illustrated in Table 6.

**Table 6:** Univariate regression analysis to identify the predictors for MASEI scores among HD patients (*n* = 41)

Predictor variable	MASEI-inflammatory		MASEI-damage		Total MASEI	
	B	P	B	P	B	P
Age (years)	0.094	0.042*	0.062	0.357	0.156	0.086
Duration of HD	0.157	0.485	0.875	0.004*	1.032	0.014*
Hemoglobin level (g/dl)	−0.001	0.998	−0.597	0.278	−0.597	0.43
Serum cholesterol (mg/dl)	−0.021	0.164	0.010	0.639	−0.011	0.716
Serum phosphorus (mg/dl)	0.82	0.783	0.391	0.349	0.473	0.409

*P < 0.05

## Discussion

MSK manifestations are common in patients on long-term HD, with reported frequency up to 70% [19], most of which are attributable to amyloid deposition [19]. Amyloid deposits in a variety of tissues, with a higher proclivity for bone and synovial membranes. Additionally, tendons and peripheral nerves can be impacted by this serious condition [20].

Enthesitis is a common feature of spondylarthritis [21]. However, the clinical examination and radiology used to evaluate entheses are not reliable nor accurate [22].High-resolution US is commonly employed as an imaging tool in the diagnosis of enthesopathy [23]. Subclinical entheseal involvement in HD patients has not been deeply investigated [15]. As a result, this study was carried out to identify entheseal involvement in HD patients using MASEI scoring system. Gutierrez and coworkers conducted a similar study utilizing Glasgow Ultrasound Enthesitis Scoring System (GUESS), although it does not include PD evaluation or upper limb enthesis characteristics [15].

Our study sheds new light on the topography and prevalence of entheseal involvement in HD patients. To the best of our knowledge, this is the first to use MASEI scoring system in HD patients. Six entheseal sites were scanned using both GS and PDUS. Clinical examination revealed at least one sign suggestive of enthesopathy in 69 (14.02%) of 492 entheses in 41 HD patients. Musculoskeletal US (MSUS) revealed that tendon and ligament thicknesses were significantly higher in HD patients in comparison to healthy controls. Total MASEI score was positively correlated with both age and duration of HD. Age was predictive for MASEI-inflammatory component while duration of HD was predictive for both MASEI-damage component and total MASEI score.

MSUS can effectively screen for subclinical enthesopathy [24]. Chronic subclinical enthesopathy may diminish tendon mechanical resistance, thus representing a potential risk factor for tendon rupture in the future [25]. In the current study, clinical examination revealed at least one sign suggestive of enthesopathy in 69 (14.02%) of 492 entheses in 41 HD patients, which is much lower than that detected by US (68.9%). A similar pattern of results was obtained in another study,in which, US signs of enthesopathy were detected in 50% of the scanned entheseal sites in dialysis patients [15]. A high prevalence of subclinical enthesopathy has also been recognized in Behçet’s disease [26], Sjögren syndrome [27], fibromyalgia [27], psoriasis [28] and systemic sclerosis [29].

In this study, there were statistically significant higher scores of structural tendon abnormalities, enthesis thickening, bone erosions and calcification in HD patients when compared with healthy subjects. HD patients had significantly higher total MASEI score than healthy controls, also higher MASEI-inflammatory and damage scores. Overall these findings are in accordance with findings reported by Gutierrez and colleages in a study conducted on 33 dialysis patients and 33 healthy controls adopting GUESS scoring system and demonstrated a higher prevalence of enthesopathy in dialysis patients than in healthy subjects [15]. Tendon abnormalities in the form of calcific deposition, increased thickness and abnormal peritendon tissue have also been documented in another study, in which, quadriceps and Achilles tendons were evaluated in 50 HD patients by MSUS [30]. The uremic state causes the gradual retention of a significant number of molecules known as uremic retention solutes, which are poisons that would typically be discharged by healthy kidneys. Although the precise role of toxins such indoxyl sulphate and osteoprotegerin has yet to be determined. They have the ability to alter bone metabolism in patients with renal failure as well as, most likely, collagen metabolism.

Our results demonstrate a statistically significant positive association between total MASEI score and both patients’ age and duration of HD. A similar conclusion was reached by another study [15]. Age may influence the biological milieu and tendon adaptation to mechanical loading [31]. Additionally, long-term dialysis has been linked to MSK problems, including bone cysts, amyloidosis, destructive arthropathy, carpal tunnel syndrome, and spontaneous tendon rupture [32]. Contrary to the findings of Falsetti and colleagues [33], we did not find a significant association between total MASEI score and body weight.

Tendon degeneration is often caused by metabolic disorders (e.g. diabetes, hyperuricemia, or hypercholesterolemia) [31]. Hypercholesterolemia may be associated with abnormal tendon thickness or structure [34]. However, no correlation was found between MASEI scores and total cholesterol level in our cohort.

Significant osteolytic bone resorption with osteoclasts at the point of tendon insertion has been described in hyperparathyroidism in HD patients [35]. A thickened and degenerated tendon overlying an underlying uremic disease should be recognized and treated as a more advanced form of uremic tendinopathy. Secondary hyperparathyroidism may lead to tendon rupture due to changes that occur at the tendo-osseous junction [36]. Even though, our results suggest no correlation between MASEI scores and PTH level.

Using univariate linear regression analysis, age was predictive for MASEI-inflammatory component. This is consistent with what has been found in a recent study [37], they investigated the contributing factors of US lesions of entheses in healthy subjects and found that age predicted not only inflammation, but also, damage and total US enthesopathy scores. While the ability of the tendons to compensate for stress is preserved in youth, impaired resilience of aged tissues is connected to cellular senescence and changes in extracellular matrix mechanical properties [38].

One of the strengths of this study is that it represents a comprehensive scanning of entheses at both upper and lower limbs in HD patients. Also, a control group of sex- and age-matched participants was included. However, our study has some limitations: firstly, the relatively small sample size; sample size calculation was not made according to the primary outcome measurement. Secondly, the cross-sectional nature resulting in only a single evaluation of the entheses with absence of long-term follow-up. Finally, only one sonographer was utilized; a second sonographer would aid in determining the reproducibility of findings because US is an operator-dependent examination with possible inter-observer variability. A future study’s goal would be to determine inter and intra reader variability.

To the best of our knowledge, this is the first study to use the MASEI scoring system to detect enthesopathy in HD patients. In conclusion, there is a high prevalence of asymptomatic subclinical enthesopathy in both upper and lower limbs in HD patients. Additionally, the burden of entheseal US alterations is much higher than in healthy subjects. MSUS can be helpful in the early detection of entheseal abnormalities in HD patients. The duration of HD is the significant predictor of enthesopathy in these patients. Finally, entheseal involvement in HD patients do not correlate with PTH or serum calcium level. The current findings need to be replicated in other ethnic groups and with a larger sample size.

## Data Availability

The datasets used and/or analysed during the current study available from the corresponding author on reasonable request.
