# Correction: Ultrasonographic assessment of entheseal sites of upper and lower extremities in hemodialysis patients using Madrid Sonography Enthesitis Index

**DOI:** 10.1186/s12891-022-05655-5

**Published:** 2022-08-04

**Authors:** Reham Sabry, Samar Tharwat, Mohammed Kamal Nassar, Ehab E. Eltoraby

**Affiliations:** 1 Nephrology unit, Internal Medicine Department, Mansoura New General Hospital, Mansoura University, Mansoura, Egypt; 2 Rheumatology & Immunology Unit, Internal Medicine Department, Faculty of Medicine, Mansoura University, El Gomhouria St, Mansoura, Dakahlia Governorate 35511 Egypt; 3 Department of Internal Medicine, Mansoura Nephrology & Dialysis Unit (MNDU), Faculty of Medicine, Mansoura University, Mansoura, Egypt

**Correction: BMC Musculoskelet Disord 23, 606 (2022)**

**https://doi.org/10.1186/s12891-022-05512-5**

Following the publication of the original article [1] the authors would like to correct the declared corresponding author as “Samar Tharwat” rather than “Reham Sabry”.

The original article [1] has been updated.
